# Mitochondrial Dynamics in Mitochondrial Diseases

**DOI:** 10.3390/diseases5010001

**Published:** 2016-12-23

**Authors:** Juan M. Suárez-Rivero, Marina Villanueva-Paz, Patricia de la Cruz-Ojeda, Mario de la Mata, David Cotán, Manuel Oropesa-Ávila, Isabel de Lavera, Mónica Álvarez-Córdoba, Raquel Luzón-Hidalgo, José A. Sánchez-Alcázar

**Affiliations:** Centro Andaluz de Biología del Desarrollo (CABD-CSIC-Universidad Pablo de Olavide), and Centro de Investigación Biomédica en Red Enfermedades Raras, Instituto de Salud Carlos III, Sevilla 41013, Spain; juasuariv@gmail.com (J.M.S.-R.); marvp75@gmail.com (M.V.-P.); patricia_dlcruz_ojeda@hotmail.com (P.d.l.C.-O.); mrdelamata@gmail.com (M.d.l.M.); lobolivares@hotmail.com (D.C.); manueloropesa@hotmail.com (M.O.-Á.); isadelavera@gmail.com (I.d.L.); monikalvarez11@hotmail.com (M.Á.-C.); raqueluzon@gmail.com (R.L.-H.)

**Keywords:** mitochondrial disease, mitochondrial dynamics, mitophagy, mitochondrial fusion, mitocondrial fission

## Abstract

Mitochondria are very versatile organelles in continuous fusion and fission processes in response to various cellular signals. Mitochondrial dynamics, including mitochondrial fission/fusion, movements and turnover, are essential for the mitochondrial network quality control. Alterations in mitochondrial dynamics can cause neuropathies such as Charcot-Marie-Tooth disease in which mitochondrial fusion and transport are impaired, or dominant optic atrophy which is caused by a reduced mitochondrial fusion. On the other hand, mitochondrial dysfunction in primary mitochondrial diseases promotes reactive oxygen species production that impairs its own function and dynamics, causing a continuous vicious cycle that aggravates the pathological phenotype. Mitochondrial dynamics provides a new way to understand the pathophysiology of mitochondrial disorders and other diseases related to mitochondria dysfunction such as diabetes, heart failure, or Hungtinton’s disease. The knowledge about mitochondrial dynamics also offers new therapeutics targets in mitochondrial diseases.

## 1. Introduction

Mitochondria are vital organelles for every nucleated cell that are responsible for making energy in the form of ATP by the oxidative phosphorylation (OXPHOS) system. Mitochondria are also involved in vital metabolic processes, including steroid and heme biosynthesis, intermediary metabolism, calcium and iron homeostasis, redox signalling, programmed cell death, innate immunity, and influence the regulation of complex physiological processes [1].

Due to the important functions of mitochondria in cell homeostasis, mitochondrial dysfunctions cause a great variety of diseases which can affect almost all the tissues and organs in the body [2].

In general, the term primary mitochondrial diseases refers to those inborn error disorders caused by total or partial dysfunction of the mitochondrial electron transport chain associated with the OXPHOS system [3]. The estimated prevalence of these diseases is at least one in 5000 people, although it could be higher [4].

Mitochondrial proteins are encoded by either mitochondrial DNA (mtDNA) or nuclear DNA (nDNA). Consequently, mitochondrial diseases can be caused by mutations in both genomes. This double genetic control of mitochondrial function gives rise to different patterns of inheritance of mitochondrial diseases and complicates their characterization and diagnosis. Mitochondrial diseases have a great variety of phenotypes which manifest mainly in organs and tissues with higher energy demand, such as neuronal tissue, muscle, heart, and kidney [5].

There is a huge variety in the symptoms and severity of mitochondrial diseases from life-threatening to almost asymptomatic, sometimes taking both extremes in members of the same family. Although the clinic-pathological and molecular features of mitochondrial diseases have been extensively studied in recent years, many physiopathological aspects need further research. In addition, there are no effective treatments for mitochondrial disease, although current therapies may alleviate select clinical symptoms and theoretically may offer a means of preventing disease progression [6].

On the other hand, alterations in mitochondrial function are increasingly being recognized as a secondary contributing factor in many neurological, cardiovascular and metabolic diseases [7,8].

In this review, we try to clarify the role of mitochondrial dynamics in primary mitochondrial diseases and other disorders related to mitochondrial dysfunction.

## 2. Mitochondrial Dynamics

Currently, it is not completely understood how dysfunctional mitochondria are distinguished from functional ones on a molecular level. However, it is well known that cells have developed several ways to perform mitochondrial quality control [9]. First of all, mitochondria as dynamic organelles are constantly undergoing fission and fusion to adapt to changes in the cellular environment. Mitochondrial fission is needed to create new mitochondria, but it also allows the segregation of damaged mitochondria. In contrast, mitochondrial fusion produces tubular or elongated mitochondria which allows for exchanging of material between mitochondria and may compensate their functional defects.

**Figure 1:**
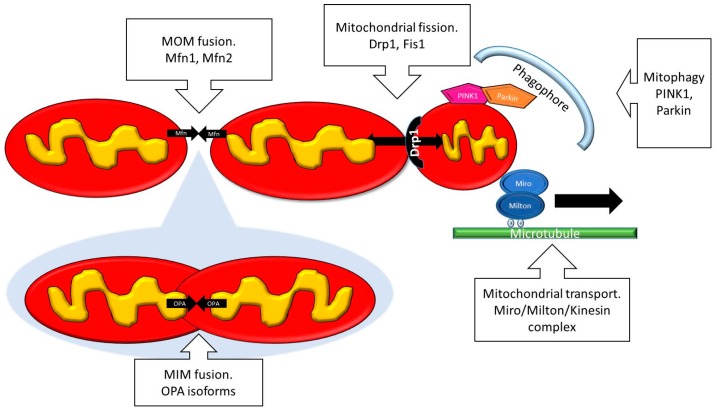
Scheme showing the main proteins implicated in mitochondrial dynamics. MOM: Mitochondrial outer membrane; MIM: Mitochondrial inner membrane.

In physiological conditions, mitochondrial fusion and fission occur in a constant and balanced manner to adapt the morphology of the mitochondrial network to the metabolic needs of the cell (Figure 1) [10]. Healthy mitochondria form elongated tubules that continually divide and fuse to form a dynamic interconnecting network. The molecular machinery that mediates this organelle fission and fusion is necessary to maintain mitochondrial integrity by facilitating mtDNA and protein quality control [11]. Mitochondrial fission and fusion proteins are highly regulated by post-translational modifications such as phosphorylation, sumoylation and ubiquitination [12].

## 3. Mitochondrial Fusion

Mitochondrial fusion in mammals is mediated by Mitofusin 1 and Mitofusin 2 (Mfn1 and Mfn2) and optic atrophy 1 (OPA1) proteins. Mfn1 and Mfn2 are dynamin-related GTPases responsible for fusion of mitochondrial outer membranes (MOM). Mfn1 and Mfn2 can functionally replace each other because the induction of Mfn2 overexpression in cells deprived of Mfn1 completely abolished the effect of the absence of Mfn1 [13]. In humans, mutations in Mnf2 can cause neurodegenerative disease such as Charcot-Marie-Tooth Neuropathy type 2A [14], suggesting that it is necessary to preserve the correct function of mitochondrial fusion to satisfy the energy demands of the cell.

OPA1 is also a dynamin-related GTPase, which is responsible for fusion of mitochondrial inner membranes (MIM) [15]. The OPA1 protein is associated with different functions, such as maintenance of the respiratory chain and membrane potential, cristae organization and control of apoptosis, as well as mtDNA maintenance [16]. Normal functioning of OPA1 requires the presence of Mfn1 but not of Mfn2 [17]. Dominant optic atrophy (DOA), a neuro-ophthalmic condition characterized by a bilateral degeneration of the optic nerves, is associated with mutations in nuclear genes encoding mitochondrial proteins, primarily the OPA1 gene [18]. Furthermore, heterozygous OPA1 mutations are also associated with a broad range of extra-ocular symptoms including ataxia, sensorineural deafness, chronic progressive external ophthalmoplegia, axonal sensory-motor polyneuropathy, and mitochondrial myopathy [18,19,20].

Once imported into mitochondria, OPA1 can be proteolytic cleavage in two isoforms: long and short [21]. The short isoform is the result of the Oma1 and Yme1L protease activities which are regulated by ATP levels and oxidative stress [22,23,24]. Under physiological conditions, both long and short isoforms are generated in equal amounts and their imbalance affects the OPA1-mediated MIM fusion mechanism [25]. The balance between both isoforms allows mitochondria to adapt its fusion rate in several metabolic conditions [26].

The fusion process is critical for maintenance of mitochondrial function, as a blockage of mitochondrial fusion results in a loss of mitochondrial membrane potential [13]. Furthermore, mitochondrial fusion allows the spreading of molecules and mtDNA throughout the entire mitochondrial compartment. This serves to optimize mitochondrial function and avoid the accumulation of mitochondrial mutations during aging. Furthermore, it has been proposed that mitochondrial fusion can be considered as a “rescue” mechanism that prevents the elimination of damaged mitochondria by mitophagy [27].

## 4. Mitochondrial Fission

On the other hand, mitochondrial fission plays an important role in mitochondrial replication and the removal of damaged organelles by selective autophagy. First, mitochondria are replicated by mitochondrial fission during cell division to ensure that daughter cells contain as many mitochondria as their mother cell. On the other hand, as soon as the mitochondrial network becomes dysfunctional, fission events allow the spatial isolation of damaged mitochondria. During mitochondrial fission, the dynamin-related GTPase dynamin-related protein 1 (Drp1) is recruited from the cytosol onto the mitochondrial outer membrane. There, Drp1 complexes constrict the mitochondrial tubule to mediate fission [28]. A key mechanistic process is how Drp1 is recruited to the mitochondrial surface. In mammals, several integral membrane proteins of the mitochondrial outer membrane—mitochondrial fission protein 1 (Fis1), mitochondrial fission factor (Mff), and mitochondrial dynamics proteins of 49 and 51 kDa (MiD49 and MiD51, respectively)—have been proposed to act as receptors that recruit Drp1 to the mitochondrial surface [29].

The mitochondrial fission-promoting activity of Drp1 is controlled by the phosphorylation of Drp1 at Ser637 [30]. PKA, cAMP-dependent protein kinase, phosphorylates Drp1 and inhibits the translocation of Drp1 to mitochondrial fission sites. Conversely, the calcineurin-mediated dephosphorylation of Drp1 results in the recruitment of Drp1 to the mitochondria and promotes mitochondrial fission.

Just one case of neonatal lethality has been linked to a defect mutation in Drp1; the defect was associated with a severe defect in the fission of both mitochondria and peroxisomes [31]. Clinically, the patient presented microcephaly, abnormal brain development, optic atrophy and hypoplasia, persistent lactic acidemia, and a mildly elevated plasma concentration of very-long-chain fatty acids.

As mitochondrial fission is critical for mitochondrial division, size, and shape, and for the distribution of mitochondria throughout the cell, especially in neurons, Drp1 plays an essential role in the pathophysiology of several neurodegenerative diseases such as Parkinson’s disease [32] or Huntington’s disease [33].

## 5. Mitochondrial Transport

Mitochondrial mobility through the cytoskeleton is very important for the mitochondrial network quality control. Mitochondria make use of the kinesin/dynein motor to move along the microtubules. The attachment between the mitochondria and microtubules is performed by the interaction between a mitochondrial membrane protein (Miro) and an adapter protein (Milton) [34]. Thus, motility of the mitochondria depends on the kinesin/Miro/Milton complex and its regulation allows the organelles to be delivered to areas of interest. Although it is not clear if mitochondrial motility prevents mitophagy, it has been demonstrated that knockdown of Miro promotes the perinuclear clustering of mitochondria and accelerates mitochondrial removal by mitophagy [35]. Moreover, Parkin also ubiquitinates Miro, preventing mitochondrial movement and facilitating mitochondrial isolation [32].

Although, Miro and Milton have not been directly implicated in a specific genetic disease, altered mitochondrial transport has been related to motor neuron diseases characterized by progressive axonal degeneration [36]. Indeed, mitochondria must be positioned properly to serve the needs of the cell. Thus, mitochondria are delivered to areas of the axon where metabolic demand is high, such as synapses [37], active cones and branches [38] or regions of axonal protein synthesis [39]. Some examples of diseases in which mitochondrial transport has been associated with pathophysiological alterations are Parkinson’s disease, Alzheimer’s disease, amyotrophic lateral sclerosis and schizophrenia [40].

Defects in both fusion and fission have been shown to decrease mitochondrial movement, and conversely, transport defects affect mitochondrial morphology [41]. However, the mechanisms underlying this interplay remain to be determined.

## 6. Mitophagy

Mitophagy is the process by which dysfunctional or damaged mitochondria are selectively targeted by autophagosomes and delivered to lysosomes to be recycled by the cell. Mitochondrial homeostasis requires a perfect equilibrium between mitophagy and mitochondrial biogenesis. Extensive mitophagy can lead to bioenergetic failure whereas excessive mitochondrial biogenesis can generate detrimental levels of reactive oxygen species (ROS) and promote apoptosis [42]. Consequently, maintenance of a balanced healthy mitochondrial population by both processes is essential for cellular function and survival [43,44,45].

The most characterized mechanism regulating the recruitment of autophagosomes to mitochondria is that driven by phosphatase and tensin homolog (PTEN)-induced putative kinase 1 (PINK1) and Parkin [46]. Mutations in the PINK1 and Parkin genes are well known causes of autosomal recessive forms of Parkinson’s disease, and numerous studies link this to the role of these proteins in mitochondrial quality control [47].

Other less known mechanisms related to mitochondrial quality control have been suggested, such as Parkin-independent mitophagic mechanisms, or mitochondrial spheroids formation [15]. However, further investigation is needed to understand the importance of these mechanisms in mitochondrial turnover.

Since mitochondrial fission may be necessary for the initiation of mitophagy, ubiquitination of Mfn2 by Parkin could be important for targeting dysfunctional mitochondria to mitophagy [48]. In fact, it has been hypothesized that Parkin facilitates mitophagy by eliminating Mitofusin and increasing the fission process, thus there is an increased number of small and isolated mitochondria which can be easier handled by the phagosome. However, mitochondrial fission would be necessary but not sufficient for mitophagy activation because mitochondria have to be additionally dysfunctional and/or depolarized to prevent mitochondrial fusion and induce mitophagy [49].

## 7. Mitochondrial Diseases and Mitochondrial Dynamics

**Figure 2:**
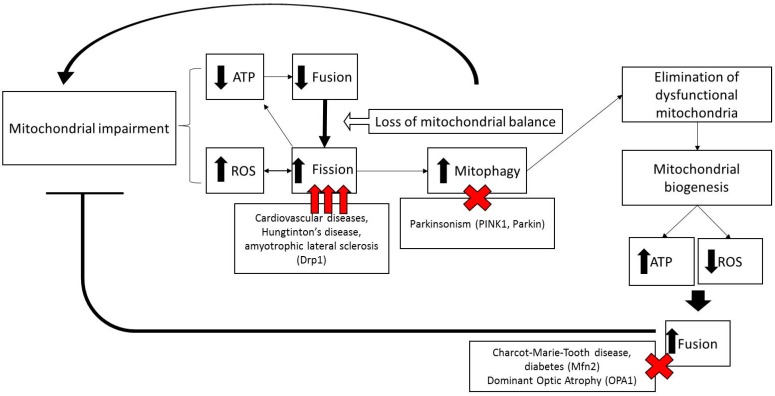
Scheme illustrating the role of mitochondrial dynamics in mitochondrial diseases. Mitochondrial dysfunction and reactive oxygen species (ROS) production induces decreased mitochondrial fusion and mitochondrial fragmentation and mitophagy. Elimination of dysfunctional mitochondria, associated to increased mitochondrial biogenesis, restores ATP levels, decreases ROS production and increases mitochondrial fusion and function.

All mitochondrial fission and fusion proteins are nuclear-encoded [50]. Generally, fission allows segregation of damaged mitochondria and fusion grants material exchange between functional mitochondria and damaged mitochondria, establishing the integrity of the entire mitochondrial network. Therefore, alterations in mitochondrial dynamics often play a crucial role in mitochondrial disorders (Figure 2).

Primary mitochondrial disorders represent a diverse group of diseases associated with dysfunction of the mitochondrial respiratory chain due to mutations in nDNA or mtDNA. The presentation usually depends on generalized or tissue-specific decreases in ATP production. In addition, the vast majority of mitochondrial diseases are characterized by an increased ROS production leading to oxidative stress. Increased mitochondrial ROS production includes damage of mitochondrial and/or nuclear DNA and RNA [51], protein [52] and lipid [53] oxidation. Some mitochondrial disorders affect a single organ but many affect most of them. The range of symptoms of mitochondrial diseases is broad and includes neuropathies, myopathies, cardiovascular disorders and gastrointestinal, endocrine and hematological alterations [54].

It is not clear if alterations in the mitochondrial network could promote ROS production. It has been reported that oxidative stress initiates mitochondrial fission in neurons and that Mfn2 expression was protective [55]. On the other hand, it has been shown that the Drp1-mediated fission accompanies respiration increase and ROS production induced by a high level of glucose [56]. In fact, cells exposed to extracellular or intracellular ROS were shown to have impaired morphology of the mitochondrial network, depending on the load of ROS and duration of the treatment [57].

Since most of the proteins involved in mitochondrial dynamics are GTPases, morphology of the mitochondrial network may change depending on the local GTP gradients and the mitochondrial energetics [58]. Generally, GTP levels are proportional to ATP levels, because GTP is formed from ATP by matrix and cytosolic nucleoside diphosphate kinases. During fission, Drp1 is recruited towards the outer membrane proximity depending on the local cellular GTP levels and information signaling. In the same way, if OPA1 senses elevation of local GTP levels, the OPA1-mediated fusion may dominate. In contrast, a decrease of GTP levels may suppress mitochondrial fusion and mitochondrial fission would prevail [59].

Autophagy and selective autophagy are induced in response to different energetic or oxidative stresses [60]. Autophagy and/or mitophagy have been demonstrated to be misregulated in several cellular and animal models of mitochondrial diseases [61,62,63]. Furthermore, mitophagy may play an essential role in diseases caused by mtDNA mutations by preventing heteroplasmy expansion [64]. Heteroplasmy is the coexistence between wild mtDNA and mutant mtDNA particles in the same cell. Commonly, high levels mutant mtDNA increase the severity of the disease. It is also known that multiple deletions in mtDNA may accumulate in some cases of dominant optic atrophy syndromes associated with OPA1 mutations [19,65]. Thus, it is assumed that the balance between fusion and fission is necessary to mix mtDNA genomes and promote the elimination of mitochondria with excessive mutant mtDNA via mitophagy. Alterations in these processes could favor the expansion of mutant mtDNA under specific pathological conditions. The critical role of mitochondrial dynamics in mtDNA maintenance has been clearly established in a mouse model of impaired mitochondrial fusion with conditional deletion of Mfn1 and Mfn2 in skeletal muscle [66].

Both mtDNA depletion and accumulation of mtDNA mutation can be the result of impaired mitochondrial fusion, indicating the critical role of this process in mtDNA maintenance. Further evidence recently came from pathologies where Mfn2 mutations led to the accumulation of mutant mtDNA in skeletal muscle [67].

### 7.1. Diseases Caused by Mutations in Mitochondrial Dynamics Machinery

#### 7.1.1. Charcot-Marie-Tooth Disease

Charcot-Marie-Tooth (CMT) disease represents a group of clinically and genetically heterogeneous inherited neuropathies affecting the peripheral nervous system. Nowadays, there are three main forms of axonal CMTs that have been described: dominant (CMT2), recessive (CMT4C) and recessive X-linked (CMTX). CMT2A is the most common type of axonal dominant forms. Typical symptoms of CMT2A are progressive distal limb muscle weakness and/or atrophy, stepping gait, distal sensory loss, and mobility impairment.

Several studies have identified mutations in the mitochondrial fusion related Mfn2 gene as being responsible for CMT2A [68,69]. However, current models also propose that a mitochondrial transport defect could be the cause of CMT2A. Mitochondrial distribution and transport are essential for neurons because of their polarity with long axons and dendrites, and high energy demand in synaptic or growing regions. Although Mfn2 plays a crucial role in mitochondrial fusion, it has also been reported that Mfn2 interacts with the Milton/Miro/kinesin complex in neurons [70]. It has been speculated that Mfn2 could be part of a motor complex involved in both retrograde and anterograde movement of mitochondria. Thus, studies where Mfn2 was overexpressed revealed a huge aggregation of mitochondria around the nucleus [71,72]. In contrast, Mfn2 knockout models showed decreased mitochondrial transport [70]. However, mouse embryonic fibroblasts isolated from Mfn2 knockout embryos do not appear to develop major mitochondrial transport defects. In these cells, short tubular as well as round mitochondria are present. Round mitochondria lost directed movement but were still able to fuse with the mitochondrial network, resulting in mitochondria with undirected movement. In addition, round mitochondria were also able to fragment and detach from tubular mitochondria that were then able to move correctly [13]. The authors concluded that the defect in mitochondrial movement is not due to the lack of Mfn2, but rather to incorrect mitochondrial shaping induced by the depletion of Mfn2. It can also be speculated that the role of Mfn 2 in mitochondrial fusion and mitochondrial transport is a kind of quality control that would ensure that only functional mitochondria that are able to fuse would be transported [73].

#### 7.1.2. Dominant Optic Atrophy (DOA)

DOA is believed to be the most common hereditary optic neuropathy which severely impairs vision. DOA is caused by a loss of retinal ganglion cells only located in the inner retina and projecting their axons via the optic nerve to the brain. Retinal ganglion cell loss and atrophy of the optic nerve are accompanied by weakening of the optic nerve and the characteristic fundus with pallor of the optic disc [74].

Mutations in two genes—OPA1 and OPA3—and three loci—OPA4, OPA5, OPA8—are currently known for DOA. The most relevant cases are related to OPA1 mutations which affect mitochondrial fusion, energy metabolism, apoptosis, calcium levels and maintenance of mitochondrial genome integrity [75].

Genetic models that carry homozygous OPA1 mutations show embryonic lethality [76,77], but OPA1-null mouse embryonic fibroblasts can be cultured [26], which suggests an essential function of mitochondrial inner membrane fusion during development. Cell lines and patient fibroblasts with OPA1 mutations revealed a susceptibility to apoptosis and alterations of the mitochondrial respiration activity [78,79]. Based on these results, OPA1 has been proposed as a mediator between cristae remodeling and mitochondrial fusion [80]. Mitochondrial cristae remodeling and fusion are functionally distinct from each other, however they can be correlated with apoptotic cytochrome c release, which can be prevented by OPA1 overexpression [81].

In summary, the lack of OPA1 function results in a misbalance towards mitochondrial fission that leads to mitochondrial network fragmentation, impaired mitochondrial oxidative phosphorylation, increases in reactive oxygen species and altered calcium homeostasis.

## 8. Mitochondrial Dynamics in Neurodegenerative Diseases

Neuronal development requires both mitochondrial dynamic proteins Drp1 [82] and mitofusin-2 [83]. In addition, most neurodegenerative diseases show abnormal mitochondrial dynamics such as Parkinson’s disease, Huntington’s disease, and amyotrophic lateral sclerosis.

Some cases of parkinsonism are caused by autosomal recessive mutations in mitophagy related proteins, such as PINK1 [9] and Parkin [84]. Mutations in one or both proteins impair mitophagy and cause neurons to accumulate Drp1, resulting in an excessive mitochondrial fission, oxidative stress and reduced ATP production [85,86]. In Huntington’s disease, mutant huntingtin interacts with and activates Drp1, increasing mitochondrial fission in fibroblasts derived from patients [33]. Finally, in amyotrophic lateral sclerosis, there is a rare hereditable form of the disease associated with mutations of superoxide dismutase 1 that causes Drp1-mediated mitochondrial fission and neuronal death [87].

## 9. Mitochondrial Dynamics in Cardiovascular Diseases

Mitochondria occupy approximately 30% of the total cardiac cell volume in order to sustain cardiac mechanical function [88]. Emerging data suggest that the dynamics of mitochondria are relevant in different fields of cardiovascular biology such as heart development [89] and cardiomyopathies such as cardiac hypertrophy [90], heart failure [91], myocardial infarction [92] and ischemia [93].

Several studies [94,95,96,97,98] suggest a crucial role for mitochondrial fission, mainly via Drp1 expression, in pathological cardiac remodeling by abnormal expression of mitochondrial fission proteins or reduced expression of mitochondrial fusion proteins [41]. Alterations in mitochondrial dynamics can be linked to a decline in energy efficiency, as decreased mitochondrial fission is associated with a decrease in ATP production. The possible explanation is that reduced mitochondrial fission impairs appropriate mitochondrial quality control, leading to the accumulation of damaged and dysfunctional mitochondria [99]. In addition, ensuring the proper removal of dysfunctional mitochondria is critical as mitochondrial damage has been linked to diverse cardiovascular pathologies [100].

## 10. Mitochondrial Dynamics in Metabolic Diseases

Alterations in mitochondrial morphology have been found in beta-cells of patients with diabetes and diabetic animal models. Mitochondrial size is reduced in the skeletal muscle of obese and type 2 diabetic patients compared to lean subjects [101]. This reduction was detected under conditions in which no differences in the total mitochondrial volume were found between the control and obese groups, thereby indicating that the total mitochondrial mass was unaltered [102]. This phenotype has been explained by a reduced Mfn2 expression in skeletal muscle which has been detected in type 2 diabetic obese and non-obese patients compared to controls subjects [103].

It is known that Mfn2 loss-of-function disturbs glucose oxidation, oxygen consumption, mitochondrial membrane potential and proton leak in cultured cells [102,104]. This evidence suggests that Mfn2 plays a relevant role in the etiology of insulin resistance. Moreover, a positive correlation has been found between Mfn2 expression in skeletal muscle and insulin sensitivity [103].

## 11. Mitochondrial Dynamics as a Therapeutic Target of Mitochondrial Diseases and Mitochondrial Dysfunction Related Disorders

Manipulating mitochondrial dynamics is potentially an attractive approach to treat mitochondrial diseases, since shifting the equilibrium of fission and fusion may allow damaged mitochondria to be rescued by mitochondrial fusion or else be selectively eliminated by mitophagy. As soon as individual parts of the mitochondrial network become dysfunctional, fission events induced by Fis1 and Drp1, permit damaged mitochondria to become spatially isolated. Thus, dysfunctional mitochondria are distinguished on a morphological basis from the healthy mitochondria and can be presumably degraded by selective autophagy [105]. Mitochondrial fragmentation and increased mitophagy have been observed in several mitochondrial disorders.

The use of pro-autophagic agents as a therapy for different diseases is increasing in recent years. Among these agents, rapamycin and its derivatives are the most studied as therapeutic agents for the treatment of tumors [106] and neurodegenerative diseases such as Huntington’s disease or Parkinson disease [107]. Rapamycin exerts its pro-autophagic effect by competitive inhibition of mTOR (target of rapamycin) complex.

In primary mitochondrial diseases, a recent study investigating rapamycin treatment in a Leigh syndrome mouse model yielded promising results [108]. They noted a delayed onset and progression of neurological symptoms in the treated mice, with lower brain lactate and inflammation and reduced brain lesions. However, there was no change in OXPHOS capacity. The authors concluded that the improvement may have resulted from the immunosuppressive effect of the rapamycin rather than a direct effect on mitochondrial function.

Theoretically, the beneficial effects of rapamycin could be due to an increase in mitophagy [109]. Promotion of mitophagy by rapamycin has been demonstrated to be protective against the effects of rotenone, a complex I inhibitor which causes mitochondrial dysfunction [110]. In another study using human chondrocytes treated with oligomycin, autophagy induction by rapamycin treatment protected chondrocytes from oxidative stress [111].

Another strategy for mitochondrial diseases therapy is the treatment with pharmacological agents which inhibits mitochondrial fission such as mdivi-1 (mitochondrial division inhibitor 1), a Drp-1 inhibitor that selectively inhibits mitochondrial fission [59]. Recently, it has been reported that mdivi-1 treatment rescued mitochondrial morphological and functional defects induced by mutations in PINK1 [112]. Others inhibitors of the mitochondrial fission protein DRP1 such as the selective peptide inhibitor P110 could be beneficial by decreasing aberrant mitochondrial fission in these disorders [113]. Moreover, because a number of neurodegenerative diseases result from aberrant mitochondrial fusion [41], promoting fusion via a fission-inhibiting compound could have therapeutic potential. Genetic approaches have shown that inhibiting mitochondrial fusion shifts the fusion/fission equilibrium towards fission [13], and vice versa [114]. Therefore, the discovery of inhibitors of mitochondrial fusion and fission may be useful for the treatment of mitochondrial disorders, whether they are caused by dysfunctions of mitochondrial dynamics or not.

Furthermore, targeting mitochondrial dynamics can be a strategy to modulate heteroplasmy in mtDNA mutations. Thus, it has been suggested that mitochondrial fission prevents the increase of heteroplasmy in cell models of mitochondrial diseases. In fact, it has been observed that the knockdown of Drp1 causes an increase of percentage of heteroplasmy from 80% to 96% of m.3243A > G in a cell culture of rhabdomyosarcoma [114]. Overexpression of Parkin in cybrids with 80% of mutated mtDNA (cytochrome c oxidase I mutation) led to an increase in mitophagy, which allowed a reduction of the mutational load up to 26.7%, and most importantly, this reduction remained over time [64]. Moreover, cytochrome c oxidase (COX) activity was restored in cybrid cells enriched for wild-type mtDNA, indicating that a decrease of heteroplasmy could improve the pathophysiology of the disease. Therefore, upregulation of Parkin expression may be beneficial for hereditary mitochondrial diseases. These findings also indicate that endogenous Parkin levels may be a limiting factor for the negative selection of dysfunctional mitochondria in some cell types. Thus, low levels of Parkin may prevent the physiological elimination of damaged mitochondria with a high level of heteroplasmy, which are accumulated in the cells and cause the symptoms of the disease.

## 12. Conclusions

Mitochondrial dynamics are essential during embryonic development and tissue homeostasis and its alterations are underlying in several neuropathies and myopathies. Mitochondrial fusion and fission are multi-factorial processes that control mitochondrial shape and function. In addition, these opposing processes have reciprocal interactions with mitochondrial transport and mitophagy.

Taken together, the studies reviewed here clearly indicate that perturbations in mitochondrial dynamics are directly or indirectly involved in mitochondrial disorders. With the exception of direct mutations in mitochondrial fusion/fission proteins, most of the alterations in mitochondrial dynamics are secondary side effect of the OXPHOS defects associated with mitochondrial disorders. That implies that dysfunction of mitochondrial dynamics causes mitochondrial diseases and conversely, mitochondria alterations affect mitochondrial dynamics.

Manipulation of mitochondrial fusion and fission may alleviate the phenotype in mitochondrial diseases by optimizing energy production, mitophagy and ROS reduction. There are compelling reasons for envisioning that the modulation of mitochondrial dynamics will ultimately lead to new therapeutic approaches for mitochondrial diseases.
